# Biomarkers of Cardiac Dysfunction and Mortality from Community-Acquired Pneumonia in Adults

**DOI:** 10.1371/journal.pone.0062612

**Published:** 2013-05-07

**Authors:** Catherina L. Chang, Graham D. Mills, Noel C. Karalus, Lance C. Jennings, Richard Laing, David R. Murdoch, Stephen T. Chambers, Dominic Vettise, Christine M. Tuffery, Robert J. Hancox

**Affiliations:** 1 Department of Respiratory Medicine, Waikato Hospital, Hamilton, New Zealand; 2 Department of General Medicine, Waikato Hospital, Hamilton, New Zealand; 3 Canterbury Health Laboratories, Christchurch, New Zealand; 4 Department of Pathology, University of Otago, Christchurch, New Zealand; 5 Canterbury Respiratory Services, Canterbury District Health Board, Christchurch, New Zealand; 6 Department of Preventive and Social Medicine, University of Otago, Dunedin, New Zealand; Universidade Federal do Rio de Janeiro, Brazil

## Abstract

**Background:**

Cardiac dysfunction is common in acute respiratory diseases and may influence prognosis. We hypothesised that blood levels of N-terminal B-type natriuretic peptide (NT-proBNP) and high-sensitivity Troponin T would predict mortality in adults with community-acquired pneumonia.

**Methods and Findings:**

A prospective cohort of 474 consecutive patients admitted with community-acquired pneumonia to two New Zealand hospitals over one year. Blood taken on admission was available for 453 patients and was analysed for NT-proBNP and Troponin T. Elevated levels of NT-proBNP (>220 pmol/L) were present in 148 (33%) and 86 (19%) of these patients respectively. Among the 26 patients who died within 30 days of admission, 23 (89%) had a raised NT-proBNP and 14 (53%) had a raised Troponin T level on admission compared to 125 (29%) and 72 (17%) of the 427 who survived (p values<0.001). Both NT-proBNP and Troponin T predicted 30-day mortality in age-adjusted analysis but after mutual adjustment for the other cardiac biomarker and the Pneumonia Severity Index, a raised N-terminal pro-brain natriuretic peptide remained a predictor of 30-day mortality (OR = 5.3, 95% CI 1.4–19.8, p = 0.013) but Troponin T did not (OR = 1.3, 95% CI 0.5–3.2, p = 0.630). The areas under the receiver-operating curves to predict 30-day mortality were similar for NT-proBNP (0.88) and the Pneumonia Severity Index (0.87).

**Conclusions:**

Elevated N-terminal B-type natriuretic peptide is a strong predictor of mortality from community-acquired pneumonia independent of clinical prognostic indicators. The pathophysiological basis for this is unknown but suggests that cardiac involvement may be an under-recognised determinant of outcome in pneumonia and may require a different approach to treatment. In the meantime, measurement of B-type natriuretic peptides may help to assess prognosis.

## Introduction

Community-acquired pneumonia is a common reason for hospital admission and despite antibiotic treatment, patients have a substantial risk of dying [1], [2]. Clinical risk scores have been developed for adults and are widely used to identify high risk patients in need of intensive treatment and careful monitoring, and low risk patients who may be suitable for out-patient treatment [3]–[7]. These scores use clinical indicators from multiple organ systems, acknowledging the importance of systemic illness severity, advanced age, and reduced physiological reserve in determining mortality from pneumonia. For example, only four of the 20 measures in the Pneumonia Severity Index, and one of five components of the widely used CURB65 score (Confusion, Urea, Respiratory rate, Blood pressure, and age ≥65 years) are direct measures of respiratory function [4], [5].

Recent studies indicate that cardiac complications are common in patients with community-acquired pneumonia, are associated with more severe disease, and may predict prognosis [8]–[12]. Elevated levels of B-type natriuretic peptides (markers of cardiac failure due to pressure or volume overload) are reported to be common in community-acquired lower respiratory tract infections and are associated with a higher risk of adverse outcome [13], [14]. Elevated levels of cardiac troponins (indicating myocardial injury) have also been linked to increased disease severity in community-acquired pneumonia [15], [16]. We recently found that elevated levels of cardiac troponins and N-terminal B-type natriuretic peptide (NT-proBNP) had an additive effect on predicting mortality in exacerbations of COPD [17]. As far as we are aware, no studies have measured both of these markers of cardiac dysfunction in community-acquired pneumonia.

It is important to understand the role of cardiac dysfunction in community-acquired pneumonia. Not only might this help clinicians assess risk of death and institute the appropriate level of care, but it may also help to explain mechanisms of death and guide other treatment [8], [11]. We analysed high-sensitivity Troponin T and NT-proBNP levels in an existing cohort of patients admitted to hospital with community-acquired pneumonia in two New Zealand centres. We hypothesized that biochemical evidence of cardiac dysfunction and injury would be associated with a greater mortality independently of existing clinical risk prediction scores.

## Methods

### Ethics Statement

All patients gave written informed consent and ethical approvals were obtained from the Canterbury Ethics Committee and the Waikato Ethics Committee.

The Christchurch and Waikato pneumonia cohort prospectively enrolled consecutive adult patients with community-acquired pneumonia admitted to two large New Zealand district general hospitals from July 1999 to July 2000. Detailed methods are described elsewhere [5], [18]. All patients had an acute illness with clinical features of pneumonia and radiographic pulmonary shadowing involving at least one segment, which was not pre-existing or of other known cause. Chest radiographs were reviewed by a designated radiologist in each centre to confirm entry criteria and to document the extent of consolidation and associated abnormalities. Patients were excluded if: i) pneumonia was not the main reason for admission; ii) pneumonia was associated with bronchial obstruction, bronchiectasis, or tuberculosis; iii) they were severely immunocompromised with neutropenia, HIV infection, or currently receiving cancer chemotherapy; or iv) they had been hospitalized within the previous 14 days or transferred from a long-term hospital-level care facility. Clinical management was not influenced by participation in the study and followed standardised protocols based on international guidelines.

All patients with suspected or confirmed pneumonia were seen within 24 hours of admission (median time 16 hours) by a study investigator, who also collected the blood sample. Disease severity was graded using the CURB65 score and the Pneumonia Severity Index [4], [5]. Follow-up was arranged for all surviving patients at 6 weeks after admission for clinical assessment and chest radiography. Long-term survival or date of death was determined by national health database enquiry.

Plasma samples were stored at -80°C until batch analysis at Waikato Hospital laboratory with no freeze-thaw cycles. NT-proBNP and high sensitivity Troponin T levels were determined by quantitative electrochemiluminescence (Elecsys cobas e601; Roche Diagnostics Corporation, IN, USA).

The primary end-point was mortality at 30 days after admission. We also analysed mortality 1 year and 10 years after admission. High values of NT-proBNP and Troponin T were defined as greater than 220 pmol/L and 50 ng/L according to local laboratory reference values. Preliminary analysis found that the Pneumonia Severity Index was a better predictor of mortality than the CURB65 score and this was used in the subsequent multivariate analyses.

Associations between NT-proBNP, Troponin T, and the Pneumonia Severity Index were analysed using Spearman’s non-parametric correlations. Logistic regression was used to assess the associations between 30-day mortality and each cardiac biomarker with adjustment for age. Subsequent analyses included both biomarkers and the Pneumonia Severity Index in the model. Age was not included as a separate predictor in the multiply-adjusted models because it is included in the Pneumonia Severity Index. Further analyses included age*biomarker interaction terms and assessed the effects of combinations of the biomarkers. The Pneumonia Severity Index includes a measure of renal function, but because both NT-proBNP, Troponin T may be elevated due to impaired renal clearance, an additional analysis also adjusted for serum creatinine.

The sensitivities and specificities of the cardiac biomarkers and the Pneumonia Severity Index to predict 30-day mortality were assessed using receiver-operator curves (ROC). Analyses used STATA 10 (College Station, TX). P-values <0.05 were considered statistically significant.

## Results

545 patients met inclusion criteria of whom 474 (87%) consented to participate. Survival status and/or date of death were available for all patients at 30 days and 464 patients (98%) at 10 years. The median hospital stay was 5.0 days (mean 6.7 days). Sixteen (3%) patients required admission to the intensive care unit, 10 of whom required invasive ventilation. The overall in-hospital and 30-day mortality were 4.0% (19/474) and 5.7% (27/474) respectively. 262 of 474 patients (55%) had a probable microbial cause of pneumonia identified, most of which were bacterial. A viral diagnosis was made in 71 (15%) of patients and more than one organism was diagnosed in 44 patients (9%).

NT-proBNP and Troponin T values were available for 453 of 474 patients (96%). 21 patients had missing or insufficient blood samples for both assays. These patients did not differ in terms of age, sex, centre of enrolment, CURB65 score, Pneumonia Severity Index, or 30-day mortality from the remainder of the cohort (all p values >0.1). Patient characteristics are shown in Table 1. Neither NT-proBNP nor Troponin T were normally distributed and although NT-proBNP approximated to a normal distribution after log-transformation, Troponin T did not. We therefore used high vs. low values of both biomarkers in our primary analyses. Repeating the analysis using continuous log-transformed values of NT-proBNP and Troponin T provided similar findings (not shown).

**Table 1:** Cohort Characteristics.

	All patientsN = 453	Died within 30 daysN = 26	Alive at 30 daysN = 427	p-value
**Sex**				
Men (%)	233 (51)	11 (42)	223 (58)	0.420
**Ethnicity** (%)				
NZ European	379 (84)	26 (96)	355 (83)	
Maori/Pacific	61 (13)	0 (0)	61 (14)	
Other	13 (3)	1 (4)	12 (3)	<0.001
**Smoking Status** (%)				
Current	92 (20)	3 (12)	89 (21)	
Ex-smoker	210 (46)	14 (54)	196 (46)	
Never	151 (33)	9 (35)	142 (33)	0.523
**Age** at admission				
Median (IQR)	69 (51–79)	83 (73–87)	68 (50–78)	<0.001
≥65 years, n (%)	264 (58)	25 (96)	239 (56)	<0.001
**Co-morbidity**				
Chronic lung disease	171	13	158	0.212
Heart Failure	94	16	78	<0.001
Diabetes	54	3	51	0.998
Cerebrovascular disease	52	1	51	0.341
Renal disease	28	7	21	<0.001
Malignancies	19	1	18	1.000
Liver disease	6	1	5	0.299
**Pneumonia Severity Index**				
I	69	0	69	
II	65	0	65	
III	90	0	90	
IV	153	7	146	
V	77	19	58	<0.001
**CURB65 score**				
0	79	0	79	
1	114	0	114	
2	122	10	112	
3	74	8	66	
4	23	5	17	
5	1	1	0	<0.001
**NT-proBNP**, pmol/L				
Median (IQR)	91 (27–350)	1400 (530–2600)	77 (22–290)	<0.001
>220 pmol/L, N (%)	148 (33)	23 (89)	125 (29)	<0.001
**Troponin T**, ng/L				
Median (IQR)	14 (4–35)	61 (11–255)	13 (4–32)	<0.001
>50 ng/L, N (%)	86 (19)	14 (54)	72 (17)	<0.001
**% O_2_sat on Air**, median (IQR)	93 (91–96)	88 (83–91)	94 (91–96)	<0.001

Differences between patients who died and those who survived were assessed by chi-squared for categorical data and Wilcoxon rank-sum tests for continuous data. IQR = inter quartile range.

**Figure 1:**
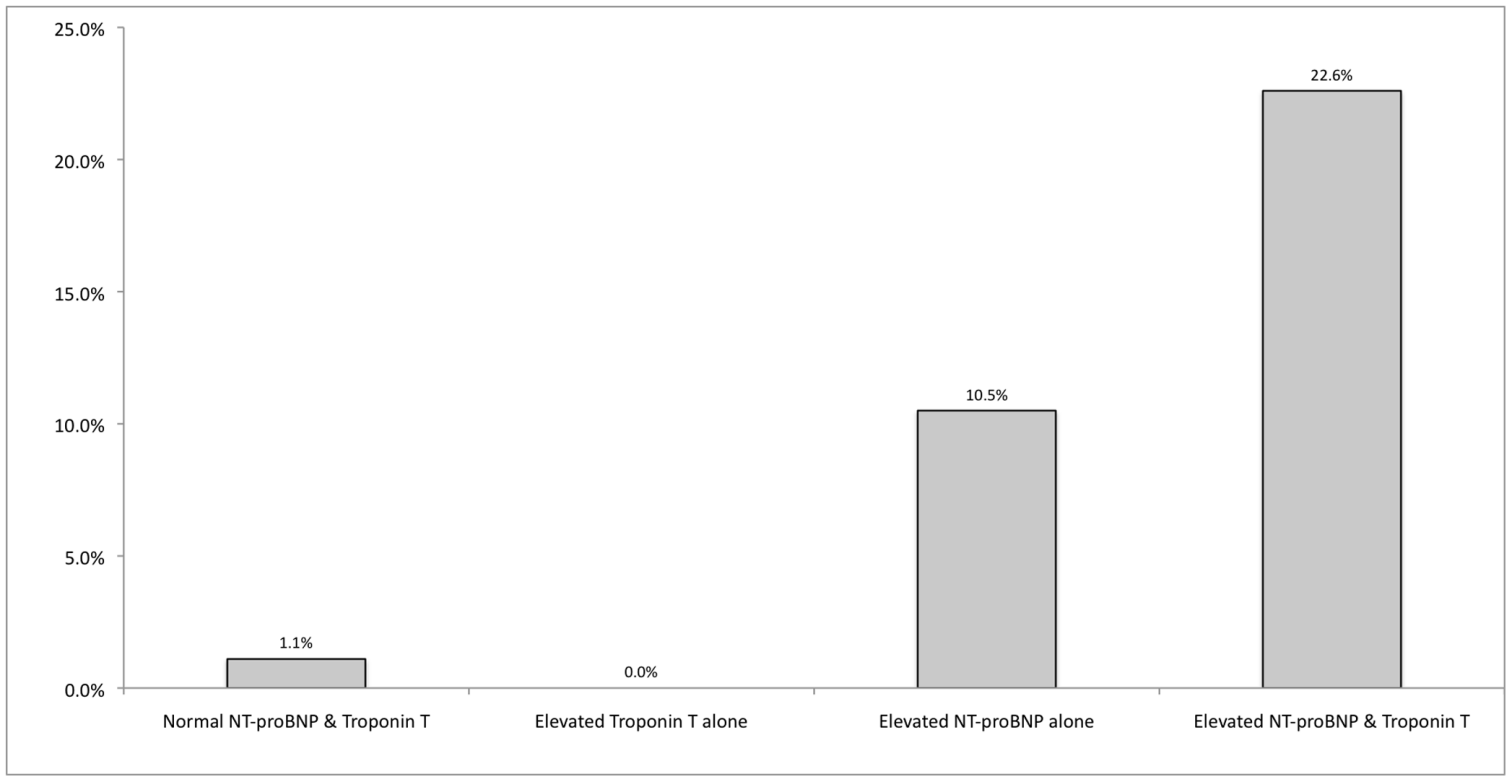
30-day mortality according to biomarker levels on admission. Mortality was lower in patients with normal NT-proBNP and Troponin T levels than patients with elevated NT-proBNP alone (9/86, p = 0.0002) and both elevated NT-proBNP and Troponin T (14/62, p<0.0001).

High values of both NT-proBNP and Troponin T were associated with 30-day mortality in age-adjusted analyses (Table 2). After mutual adjustment for the other cardiac biomarker and the Pneumonia Severity Index, NT-proBNP remained a statistically significant predictor of 30-day mortality but Troponin T did not. NT-proBNP and Troponin T values were moderately correlated (rho = 0.74, p<0.001). Few patients (n = 24) had high values of Troponin T but normal values of NT-proBNP and there were no deaths within 30 days in this category. Among patients with high values of NT-proBNP, there was a higher mortality among those who also had raised Troponin T levels (p = 0.049)(Figure 1). However, this association was not significant after adjustment for age or the Pneumonia Severity Index (p values >0.2).

**Table 2:** Logistic regression analyses of cardiac biomarkers for 30-day mortality.

	OR	95% CI	P
Age-adjusted			
High NT-proBNP	7.6	2.1–27.1	0.002
High Troponin T	2.6	1.1–6.3	0.030
Multiple-adjustments			
High NT-proBNP	5.3	1.4–19.8	0.013
High Troponin T	1.3	0.5–3.2	0.630
PSI* class	6.3	2.6–15.1	<0.001

* PSI = Pneumonia Severity Index.

Age-adjusted analyses analysed NT-proBNP and Troponin T separately with adjustment for patient age. Multiple-adjusted analyses include both biomarkers and the Pneumonia Severity Index class in the same model. High NT-proBNP and Troponin T are defined as >220 pmol/L and >50 ng/L respectively.

Neither the age*NT-proBNP nor the age*Troponin T interaction terms were statistically significant (p values = 0.233 and 0.808 respectively). However, only one patient under the age of 65 died within 30 days of admission. Excluding patients aged 64 years or younger (n = 190) did not materially influence the associations between high levels of NT-proBNP, Troponin T, and 30-day mortality in either the age-adjusted or the multivariable analyses.

A clinical history of cardiac failure was associated with higher NT-proBNP values and was a significant predictor of 30-day mortality in age-adjusted analysis (OR = 3.5, 95% CI = 3.4 to 17.9, p = 0.005), but was a weaker predictor than NT-proBNP and was of borderline statistical significance in analyses adjusted for both NT-proBNP and the Pneumonia Severity Index (OR 2.2, 95% CI, 0.9 to 5.5, p = 0.081). NT-proBNP remained a significant predictor of 30-day mortality (OR = 5.1 95% CI, 1.4 to 18.3, p = 0.012) when adjusted for a clinical history of heart failure and the Pneumonia Severity Index.

Both NT-proBNP and Troponin T were significantly associated with serum creatinine levels (rho = 0.42 and 0.47 respectively, p values<0.001). The Pneumonia Severity Index includes an adjustment for renal dysfunction and additional adjustment of the analyses for creatinine level did not materially alter the findings (not shown). Similarly, adjusting the analyses for diagnosed chronic obstructive pulmonary disease, smoking status, and antibiotic use in the 7 days before admission made no material difference to the findings (not shown).

**Figure 2:**
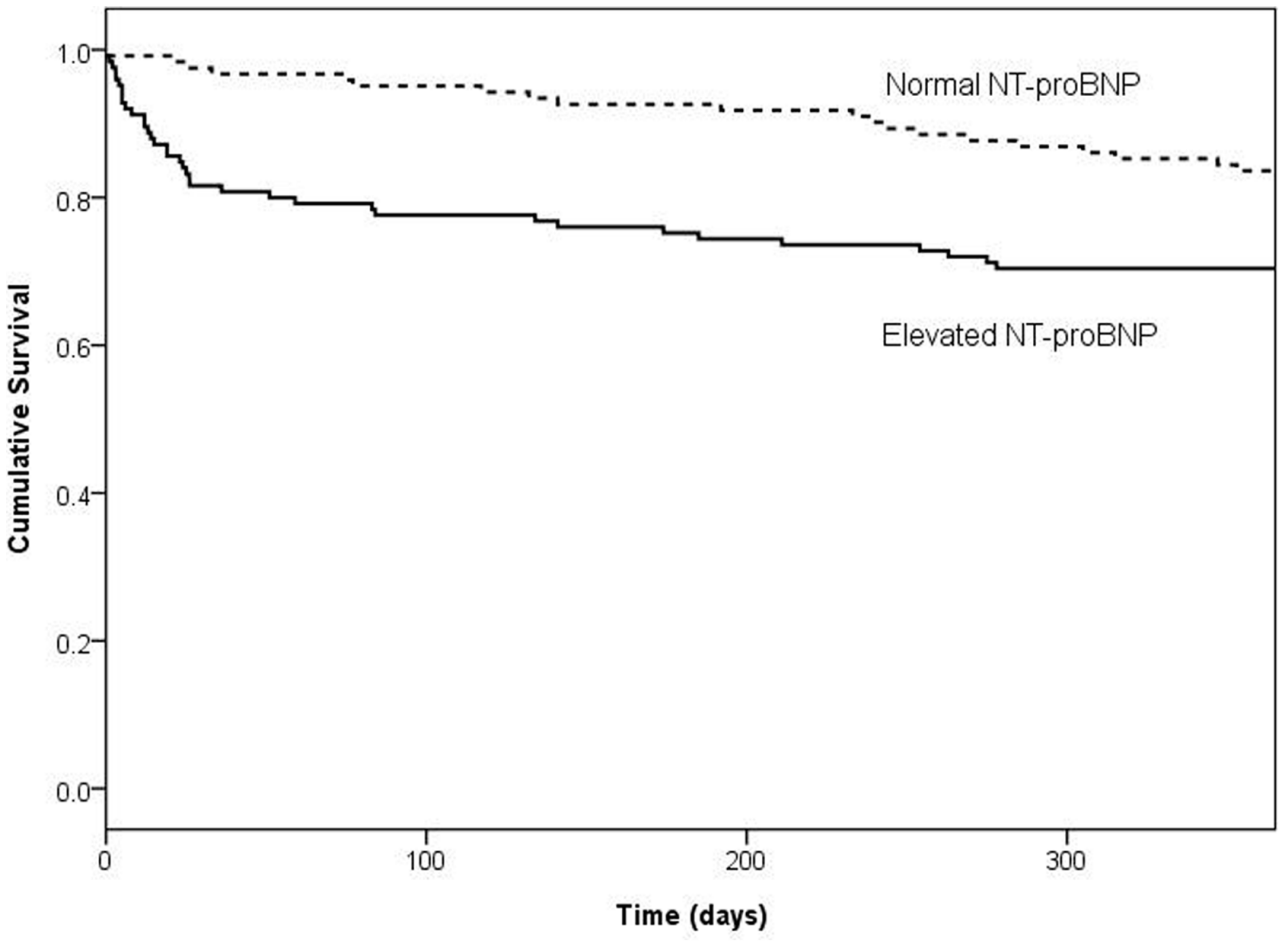
1 year Kaplan-Meier survival curve for patients following community-acquired pneumonia stratified according to NT-proBNP level. Survival was worse in patients with high NT-proBNP levels (>220 pmol/L) compared to patients with normal NT-proBNP levels (≤220 pmol/L) (log-rank test, p<0.0001).

27 patients who survived for more than one month died during the first year after admission. Neither elevated levels of NT-proBNP nor Troponin T predicted mortality between 1 month and 1 year after admission after adjusting for the Pneumonia Severity Index (OR = 1.1, 95% CI = 0.5 to 2.6, p = 0.826 and OR = 1.0, 95% CI = 0.4 to 2.6, p = 0.999 respectively) Figure 2. By contrast, the Pneumonia Severity Index was a significant predictor of mortality between 1 month and 1 year after admission (OR for each increment in category = 1.8, 95% CI 1.2 to 2.7, p = 0.004). 227/443 (51%) patients with follow-up data had died within 10 years of admission. Both NT-proBNP (OR = 2.3, 95% CI = 1.3 to 4.2, p = 0.003) and the Pneumonia Severity Index (OR 2.6, 95% CI = 2.1 to 3.3, p<0.001), but not Troponin T (OR = 1.4, 95% CI = 0.7 to 2.8, p = 0.339) predicted mortality between 1 month and 10 years after admission.

**Figure 3:**
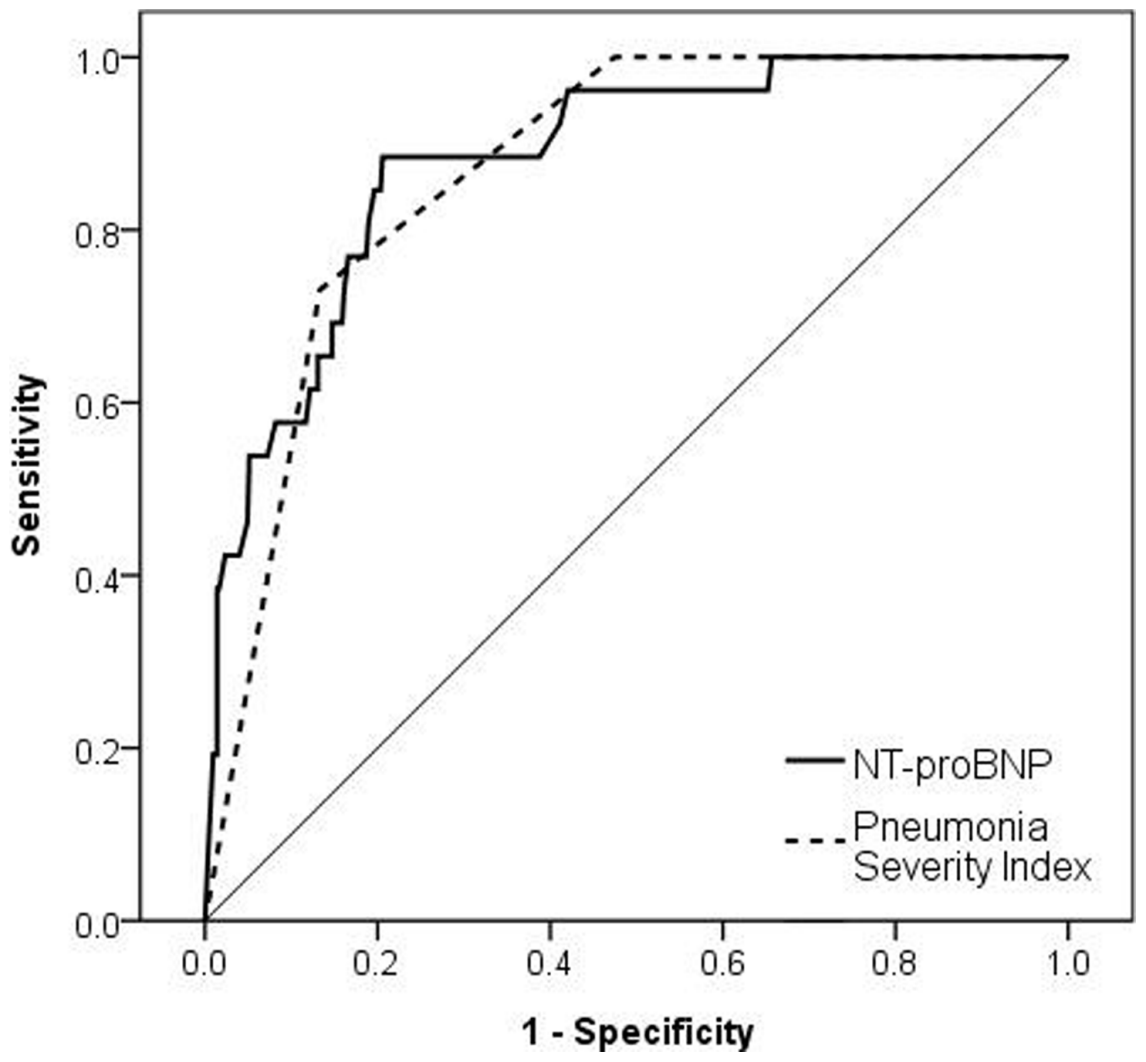
Receiver operating characteristic (ROC) curve for NT-proBNP and Pneumonia Severity Index class in 30-day mortality prediction. The area under the ROC curve = 0.8803 for NT-proBNP and 0.8701 for Pneumonia Severity Index class respectively.

ROC analyses of the sensitivity and specificity to predict 30-day mortality showed areas under the curve of 0.88 (95% CI = 0.82 to 0.94) for NT-proBNP, 0.79 (95% CI = 0.71 to 0.87) for Troponin T, and 0.87 (95% CI = 0.83 to 0.91) for the Pneumonia Severity Index (Figure 3). Our pre-specified cut point for a raised NT-proBNP of >220 pmol/L had a sensitivity of 88% and a specificity of 71% for 30-day mortality.

## Discussion

We have found that a raised level of NT-proBNP is a strong predictor of early mortality following admission to hospital for community-acquired pneumonia and that this is independent of existing clinical risk prediction scores. Elevated Troponin T was also associated with increased risk of early mortality but was not a significant predictor once clinical risk scores or NT-proBNP levels were taken into account. The findings suggest that cardiac dysfunction may be an important determinant of mortality from pneumonia and that patients without biochemical evidence of cardiac failure are at a low risk of death.

In our study the predictive value of NT-proBNP was similar to that of the Pneumonia Severity Index and out-performed the CURB65 score (data not shown). NT-proBNP predicted early mortality independently of the Pneumonia Severity Index even though the index incorporates a number of cardiovascular parameters. Age is an important predictor of mortality and is included in existing clinical risk prediction rules. We also undertook a subgroup analysis of patients over 65 years, more of whom would be expected to have elevated cardiac biomarkers, and found that even in this group, NT-proBNP was a strong and independent predictor of mortality.

Our findings are consistent with accumulating evidence that biochemical markers of cardiac dysfunction are important predictors of prognosis in acute respiratory disease [9], [10], [12]. Natriuretic peptides are known to be associated with right ventricular dysfunction and poor prognosis in pulmonary arterial hypertension, pulmonary thromboembolic disease, and undifferentiated chronic respiratory failure [19]–[24]. Similarly, elevated cardiac troponins indicate poor prognosis in patients with acute symptomatic pulmonary emboli [25], [26]. More recently, NT-proBNP and to a lesser extent, Troponin T have also been shown to predict mortality in exacerbations of COPD [17]. In pneumonia, retrospective studies and a prospective cohort of patients enrolled in a randomised trial have found that natriuretic peptides are strong predictors of outcome [14], [27], [28]. Troponin elevations are frequently noted in pneumonia and sepsis without evidence of acute coronary syndrome, although the prognostic value of troponins in pneumonia has not previously been studied [29]. Our findings support that an association between troponin levels and mortality exists but that it is not independent of natriuretic peptide levels or clinical severity indices.

Cardiac dysfunction may be an important determinant of mortality from pneumonia although the mechanism for this is unclear. Although most early deaths in our cohort were attributed to pneumonia or respiratory-related causes (data not shown), this observation needs to be interpreted with caution as the causes of death were mainly obtained from the death certificates and few patients underwent a post-mortem examination. A number of plausible mechanisms for cardiac comorbidities developing during acute pneumonia have been proposed [11]. These include an increased risk of coronary thrombosis due to the effects of systemic infection and inflammation on platelets and vascular endothelium. This mechanism would, however be expected to lead to myocardial injury and raised cardiac troponin levels. Our finding that NT-proBNP, a marker of myocardial stretch, was both a more common and a stronger indicator of prognosis than Troponin T indicates that the mechanism of cardiac involvement is not simply due to myocardial infarction or an acute coronary syndrome induced by sepsis. Sepsis itself may depress myocardial contractility at the same time as increasing myocardial work due to catecholamine release, tachycardia, and peripheral vasodilation [11], [30]. Pneumonia causes ventilation-perfusion mismatch and may result in tissue (including cardiac) hypoxia. A combination of reduced oxygen supply at the same time as increased myocardial oxygen demand could explain the association between death from pneumonia and cardiac dysfunction.

Despite extensive testing, the majority of patients did not have a confirmed microbiological diagnosis [18] making it to difficult to assess whether different causes of pneumonia differ in the extent of cardiac involvement. However, among those who did have a confirmed microbial aetiology, there were no significant differences in NT-proBNP or Troponin T levels between those with viral and bacterial diagnoses (data not shown).

A previous retrospective study has found an association between cardiac troponin levels and alveolar-arterial oxygen gradient in patients with pneumonia [15]. We found significant correlations between pulse oxygen saturations levels and both NT-proBNP and Troponin T. Lower oxygen saturations were also associated with an increased risk of 30-day mortality even after adjusting for the percent inspired oxygen (p<0.001). However, further adjustment of the multivariate analysis for oxygen saturation tended to strengthen the association between NT-proBNP and 30-day mortality, suggesting that low oxygen saturation was not the mechanism by which NT-proBNP predicted mortality. NT-proBNP also remained a strong predictor of mortality after adjustment for blood creatinine levels. Hence the association between NT-proBNP and mortality is not likely to be confounded by reduced renal clearance of NT-proBNP among patients with renal impairment.

It is unlikely that NT-proBNP is merely a marker of age or frailty. We found that, despite being strongly predictive of 30-day mortality, an elevated NT-proBNP level was not associated with subsequent deaths that occurred between one month and one year, although it did predict mortality over the next ten years. This was also true if we restricted the analysis to patients over 65 years. These observations suggest that elevated NT-proBNP levels reflect acute cardiac dysfunction in the setting of pneumonia. In contrast, the Pneumonia Severity Index includes a number of items that reflect the pre-existing health of the patient and, unsurprisingly, did predict mortality between one month and one year, as well as up to ten years after admission.

The strengths of the present study include the high recruitment rate and completeness of follow-up. The diagnosis of pneumonia was confirmed radiologically and clinical details were prospectively obtained. As NT-proBNP and Troponin T were analysed retrospectively and, since these biomarkers were not clinically available in either hospital at the time that this study was conducted, the cardiac biomarker levels could not have influenced clinical management. Samples were stored at −80°C with no freeze-thaw cycles prior to analysis. We cannot exclude the possibility that some degradation occurred during storage, but this seems unlikely because the range and levels of the cardiac biomarkers were similar to those in a recent study in which the samples were processed immediately [17]. Moreover, any degradation of the biomarkers would be expected to bias the finding towards the null and would not explain the strong association between NT-proBNP and early mortality. A limitation is that our study cohort had few deaths in those aged under 65 years making it difficult to generalise our findings to this younger patient group.

To our knowledge, this is the first time that markers of ventricular overload (NT-proBNP) and myocardial necrosis (Troponin T) have been jointly assessed in an unselected cohort of patients with pneumonia. The findings suggest that cardiac involvement is a key determinant of outcome in pneumonia. The mechanism of this association is unknown, but should prompt clinicians to make a careful assessment of cardiac function in patients with pneumonia. We do not yet know whether this should influence treatment, but measurement of NT-proBNP may help assess prognosis and identify patients at both low and high risk of death.
